# PslG-Mediated Disruption of *Pseudomonas aeruginosa* Biofilms: A Multi-Omics Investigation of Matrix Glycan Release, Metabolic Reprogramming, and Structural Disintegration

**DOI:** 10.3390/ijms27198787

**Published:** 2026-09-30

**Authors:** Yaqian Zheng, Zebin Gui, Yun Xue, You Zhou, Chaofeng Hu, Jingshuang Zhang, Luyan Z. Ma, Rui Shi

**Affiliations:** 1 Beijing Research Institute of Traumatology and Orthopaedics, Beijing 100035, China; zhengyaqian18@mails.ucas.ac.cn (Y.Z.); xueyun1102@126.com (Y.X.); zhouyou_jst@163.com (Y.Z.); zhang6jingshuang@sina.cn (J.Z.); 2 Beijing Jishuitan Hospital, Capital Medical University, Beijing 100035, China; zebin_gui0604@163.com (Z.G.); hcf1161212741@163.com (C.H.); 3 State Key Laboratory of Microbial Diversity and Innovative Utilization, Institute of Microbiology, Chinese Academy of Sciences, Beijing 100101, China; luyanma27@im.ac.cn

**Keywords:** *Pseudomonas aeruginosa*, PslG, biofilm disruption, Psl polysaccharide, glycomics, metabolomics

## Abstract

*Pseudomonas aeruginosa* biofilms are recalcitrant to conventional antibiotic therapy due to their extracellular polymeric substance (EPS) matrix, in which Psl polysaccharide serves as the primary structural scaffold. PslG glycoside hydrolase can degrade Psl and trigger biofilm dispersal, yet the dynamic glycan release profile and metabolic consequences remain poorly characterized. Mature *P. aeruginosa* PAO1 biofilms were treated with PslG (1–1000 nM) for 0–30 min. Biomass was quantified by crystal violet staining. Matrix glycan composition and untargeted metabolomics was performed using LC-MS/MS. Three-dimensional architecture was visualized by confocal laser scanning microscopy (CLSM) with SYTO 9/PI and HHA-FITC staining. PslG induced near-complete loss of Psl-specific fluorescence within 30 min. Counterintuitively, biofilm thickness decreased from 10.8 μm at baseline to approximately 3.0 μm at 5–10 min and partially recovered to 7.2 μm at 30 min, remaining below baseline; the recovery of thickness is progressively due to matrix swelling and bacterial vertical redistribution. PI staining revealed no membrane damage, confirming non-lethal dispersal. Glycomically, galactose, xylose and metabolic intermediates (glucose-6-phosphate) were enriched. Metabolomics revealed significant activation of pentose and glucuronate interconversions, pantothenate and CoA biosynthesis, carbon metabolism, and amino acid biosynthesis. The Psl cleavage mediated by PslG may trigger bacterial dispersal with vertical expansion and metabolic reprogramming toward a planktonic lifestyle. These findings provide mechanistic insights for designing Psl-targeted anti-biofilm therapeutics.

## 1. Introduction

Biofilms represent the predominant mode of bacterial existence in natural and clinical environments, serving as structured communities of sessile cells encased within a self-produced extracellular polymeric substance (EPS) matrix [1,2]. Unlike their planktonic counterparts, biofilm-embedded bacteria exhibit markedly enhanced tolerance to antimicrobial agents, host immune defenses, and environmental stressors, often by orders of magnitude [1,3]. Among biofilm-forming pathogens, *P. aeruginosa* stands out as a leading cause of persistent infections, including chronic pulmonary infections in cystic fibrosis patients, ventilator-associated pneumonia, chronic wound infections, and biofilm formation on indwelling medical devices [4]. The remarkable recalcitrance of *P. aeruginosa* infections stems primarily from its ability to form architecturally complex biofilms that effectively shield embedded cells from therapeutic intervention [5].

The structural and functional integrity of *P. aeruginosa* biofilms depends critically on the EPS matrix, which composed primarily of three distinct exopolysaccharides—Psl, Pel [6,7], and alginate [8,9]—along with extracellular DNA, proteins, and lipids [1,10]. Psl, a mannose-rich neutral polysaccharide synthesized by the *psl* operon, serves as the foundational scaffold mediating initial surface attachment, microcolony formation, and the structural maintenance of mature biofilms [11,12]. Psl fibers self-assemble into dense networks through hydrogen bonding and hydrophobic interactions, and cross-link with extracellular DNA, proteins, and Pel polysaccharide to create a protective microenvironment that facilitates nutrient trapping, antimicrobial tolerance, and cooperative bacterial behaviors [10,13,14]. Given its central role as the structural backbone, selective disruption of the Psl scaffold is considered a key strategy for compromising biofilm structural integrity.

PslG glycoside hydrolase (glycoside hydrolase family 39, GH39) is a periplasmic enzyme encoded by the *psl* operon of *P. aeruginosa*. Its periplasmic domain features a unique 32 Å-long active-site cleft that enables endo-β-glucanase-cleavage of glycosidic bonds within Psl polysaccharide chains, leading to Psl fiber depolymerization and biofilm matrix disintegration [15,16,17]. Exogenous supplementation of PslG inhibits biofilm formation and disassembles established biofilms within minutes at nanomolar concentrations, acting not through bactericidal effects but by degrading the Psl scaffold to trigger active dispersal of sessile bacteria [18]. In vivo studies have further demonstrated that PslG combined with tobramycin significantly reduces bacterial burden in a mouse wound infection model, with mechanisms involving improved antibiotic penetration, enhanced complement deposition, and promotion of host neutrophil phagocytosis and reactive oxygen species production [19,20]. Additionally, intracellular PslG has been shown to regulate Psl signaling functions, affecting c-di-GMP levels and peripheral Psl localization in daughter cells, thereby participating in the developmental regulation of biofilm microcolonies [21]. These studies have established PslG as a valuable candidate for anti-biofilm therapeutic development.

Nevertheless, most current studies rely primarily on endpoint biomass measurements—such as crystal violet staining, colony-forming unit enumeration, and macroscopic morphological observation—to assess disruption efficacy [22]. Although it can prove the effectiveness of PslG, it cannot reveal the precise chemical fingerprint of substrate degradation. It is particularly crucial that the spatiotemporal evolution of the three-dimensional structure, biofilm thickness, and bacterial spatial distribution dynamic changes in biofilms after PslG treatment have not been systematically characterized. In addition, there is still room for discussion on whether the dispersing effect of PslG accompanies bacterial death: some studies suggest that PslG releases live bacteria through physical matrix disintegration [18]. Whether the dispersed bacteria maintain membrane integrity and their redistribution patterns in three-dimensional space still need to be verified through a combination of live/dead staining and structural imaging. A multi-omics strategy integrating glycomics, metabolomics, and 3D structural imaging is expected to establish a complete mechanism chain from “molecular cleavage” to “cellular response” [23,24].

Meanwhile, PslG-mediated biofilm disruption represents not merely a physical collapse of matrix structure but likely triggers metabolic reprogramming of embedded bacteria. Released sugars may serve as carbon sources for reutilization, acidic sugar components may activate specific carbohydrate metabolic pathways, and the loss of matrix protection may trigger cellular stress adaptation responses [25]. However, metabolomic studies targeting the PslG-specific disruption process are currently lacking, and the metabolic adaptation mechanisms of cells during the continuous process of “structural collapse—nutrient release—state transition” remain unclear.

Based on the above background, this study aims to establish a multi-omics analytical framework encompassing glycomics, metabolomics, and structure to systematically dissect the dynamic process of PslG-mediated *P. aeruginosa* PAO1 biofilm disruption. Through the integration of quantitative biomass analysis, high-resolution glycan component detection, untargeted LC-MS/MS metabolomics, and three-dimensional structural imaging, this study seeks to elucidate the molecular mechanism of PslG action and provide systematic mechanistic evidence for the development of Psl-targeted anti-biofilm strategies.

## 2. Results

### 2.1. Quantitative Analysis of Biofilm Biomass Reduction

**Figure 1:**
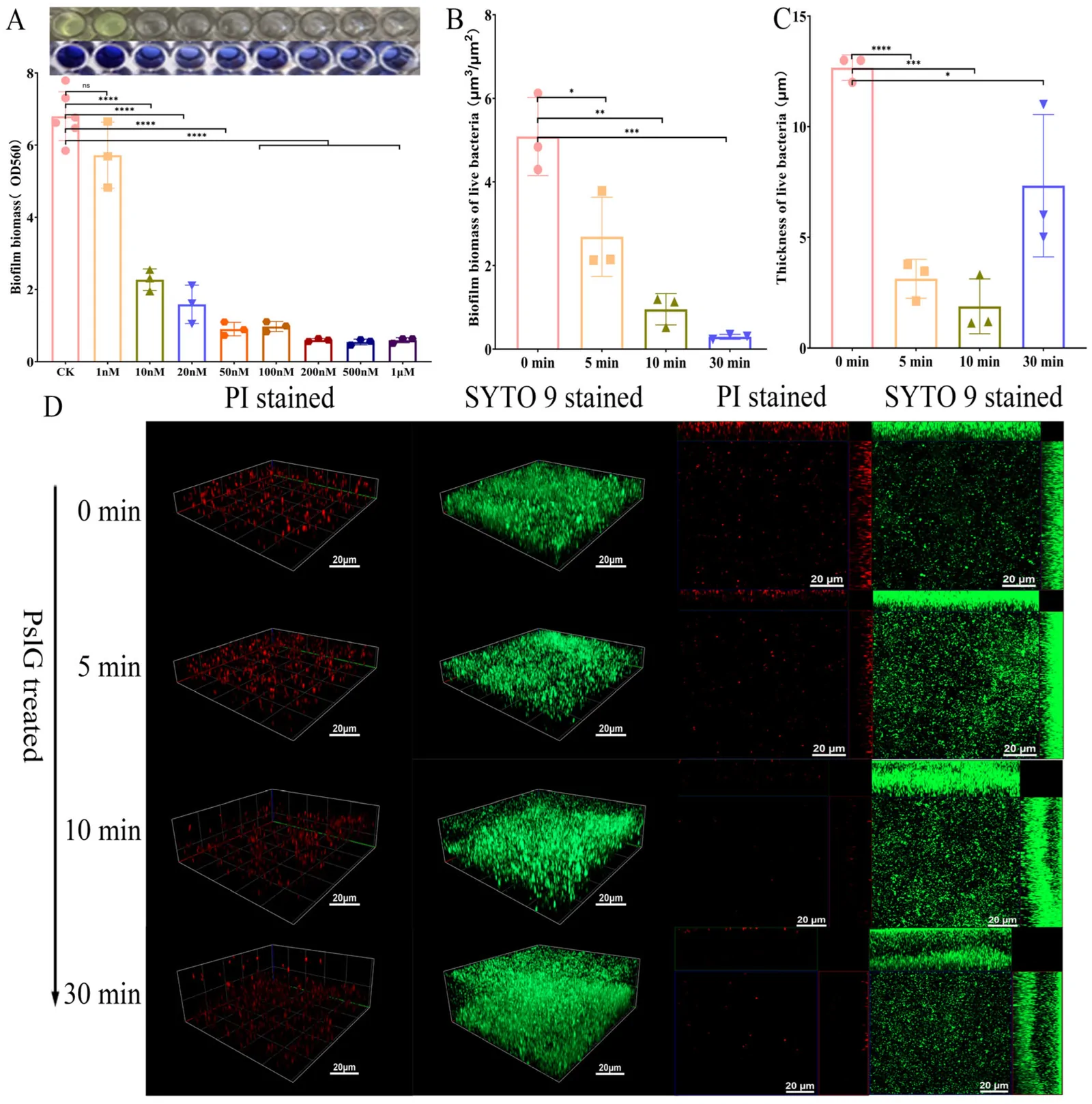
Biomass and thickness of *P. aeruginosa* biofilm treated with PslG. (**A**) The crystal violet staining results of biofilm biomass, represented by OD560; (**B**) the biomass of live bacteria within the biofilm; (**C**) the average thickness of the biofilm represented by green fluorescence stained with SYTO 9; and (**D**) the biofilm captured using CLSM. Red fluorescence stained with propidium iodide (PI) represents dead bacteria, while green fluorescence stained with SYTO 9 represents live bacteria. The left side are three-dimensional images, and the right side are top-view maximum-intensity projections. PslG treatment time ranges from 0 to 30 min. Significance was determined using Student’s *t*-test (“ns”, not significant, * *p* < 0.05; ** *p* < 0.01; *** *p* < 0.001; **** *p* < 0.0001).

A 24-hour biofilm of *P. aeruginosa* was formed by treating the bacteria with different concentrations of PslG, with a concentration of 1, 10, 20, 50, 100, 200, 500, or 1000 nM. The processing time was 30 min; results are shown in Figure 1A. At low concentrations, there was a significant residual biomass in the biofilm, while at concentrations of 50 nM and above, the biofilm biomass decreased to over 80%. The 50 nM concentration was selected for subsequent experiments as the lowest tested concentration.

We used a live/dead bacteria staining kit to stain the biofilm that has been formed for 24 h; the CLSM image results are shown in Figure 1D. The concentration of PslG used was 50 nM. As the action time increases, the biomass of live bacteria that showed green fluorescence when stained by SYTO 9 inside the biofilm gradually decreases (Figure 1B), but the average thickness of the biofilm showed a trend of first decreasing and then increasing (Figure 1C). Concurrently, SYTO 9-stained bacterial cells underwent dramatic spatial redistribution: from densely packed basal microcolonies, cells dispersed into a highly distributed, planktonic-like pattern occupying the entire vertical imaging volume, with prominent fluorescence signals detected in both the apical (upper) and basal (bottom) regions (Figure 1D). This may be the reason for the increase in thickness. The maximum-intensity projections showed a broader apparent distribution of SYTO 9-positive signal at later time points. Because top-view projections do not directly resolve vertical position, this observation is described as apparent spatial redistribution rather than direct evidence of vertical migration or a planktonic transition.

### 2.2. PslG Induces Rapid Structural Disintegration and Partial Thickness Rebound

**Figure 2:**
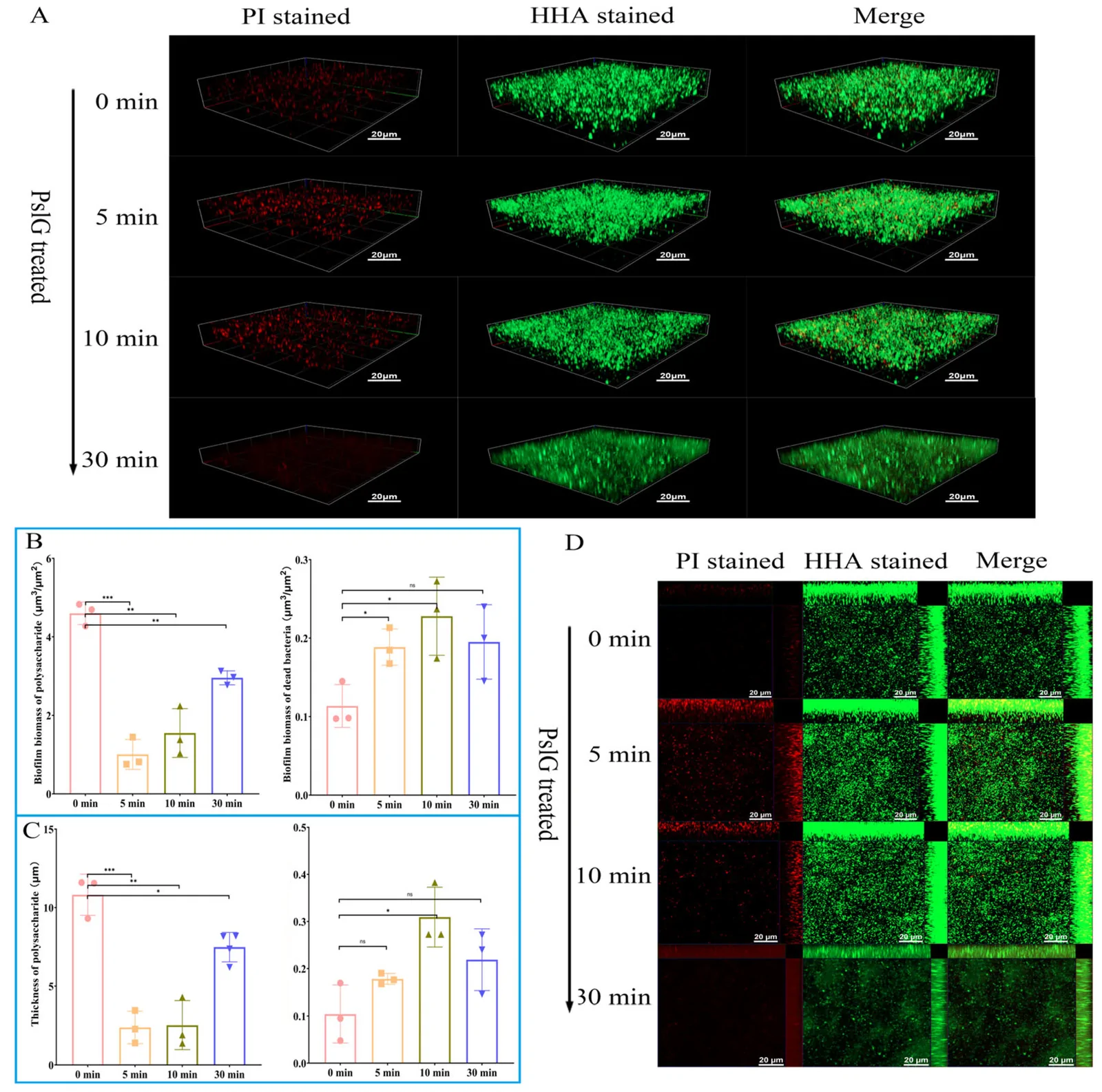
Biomass and thickness of polysaccharides and dead bacteria in *P. aeruginosa* biofilm treated with PslG. (**A**) The confocal image with PI-stained red fluorescence representing dead bacteria and HHA-FITC-stained green fluorescence representing extracellular polysaccharides; (**B**) the biomass of polysaccharides and dead bacteria in the biofilm; (**C**) the average thickness of polysaccharides and dead bacteria within the biofilm; and (**D**) top-view maximum-intensity projections of biofilm. Significance was determined using Student’s *t*-test (“ns”, not significant, * *p* < 0.05; ** *p* < 0.01; *** *p* < 0.001).

CLSM revealed profound architectural transformations (Figure 2). In untreated biofilms, HHA-FITC displayed dense Psl networks with vertical projections. PslG treatment resulted in a near-complete disappearance of HHA-FITC fluorescence within 30 min. PI dye stains dead bacteria inside biofilms, represented by red fluorescence.

Strikingly, PslG treatment resulted in a near-complete disappearance of HHA-FITC fluorescence within 30 min (Figure 2A), indicating thorough enzymatic degradation of Psl scaffolds below the detection threshold of lectin binding. Three-dimensional surface rendering visualized the transition from cohesive, mushroom-like morphology to fragmented, highly porous structures with increased vertical extension and surface area-to-volume ratio. Quantitative structural analysis revealed a progressive increase in biofilm thickness over the disruption time course: average thickness decreased from 10.8 μm at 0 min to 2.4 μm at 5 min, then increased to 2.5 μm at 10 min, and reached 7.2 μm at 30 min (Figure 2C). Similarly, in the maximum field-of-view projection, it can be observed that as the exposure time prolongs, the green fluorescence gradually diffuses to the bottom and top, and the fluorescence intensity also gradually weakens, indicating that the Psl is dispersed after degradation (Figure 2D); PI fluorescence showed no significant increase at any time point, indicating non-lethal dispersal.

### 2.3. Glycomic Profiling Reveals Sequential Depletion of Structural Sugars and Exposure of Connection Sugars

**Figure 3:**
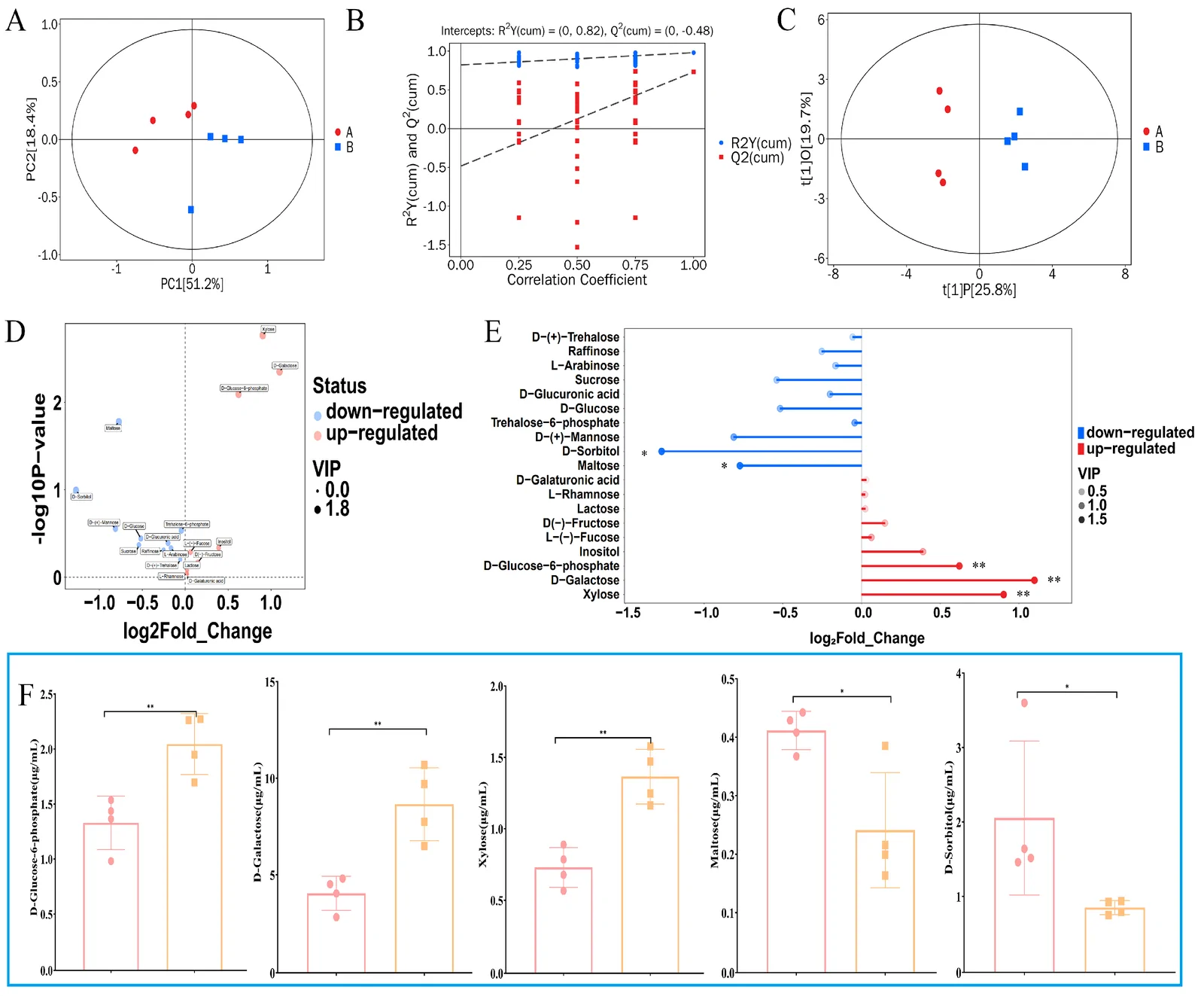
Changes in the types and contents of monosaccharides and disaccharides in the biofilm (Group A, untreated biofilm) vs. PslG treatment (Group B, the biofilm treated by 50 nM PslG for 30 min). (**A**) The principal component analysis (PCA) of QC samples, which shows the differences between two groups of samples; (**B**) the orthogonal partial least squares discriminant analysis (OPLS-DA) for orthogonal coefficients and slopes; permutation test of OPLS-DA model for group A vs. B; (**C**) score scatter plot of OPLS-DA model for group A vs. B; (**D**) a volcano plot of differential metabolites of sugars, with blue representing downregulation and red representing upregulation; (**E**) matchstick analysis for group A vs. B represents the specific name of carbohydrate differential metabolites; and (**F**) boxplot analysis for group A vs. B, the upregulated and the downregulated sugars. Significance was determined using Student’s *t*-test (* *p* < 0.05; ** *p* < 0.01).

Analysis of the 19-carbohydrate standard panel revealed distinct temporal patterns of glycan liberation and redistribution following PslG treatment (Figure 3).

The permutation test played an important role in avoiding overfitting of the test model and evaluating the statistical significance of the model. The results show that the model is stable (Figure 3B). From the OPLS-DA score plot (Figure 3C), it can be seen that the two groups of samples were significantly distinguished, and all samples were within the 95% confidence interval.

Under a provisional analysis, A1–A4 and B1–B4 were treated as four independent biological replicates, compared using *t*-tests, and adjusted across all 19 analytes. After PslG treatment, the disintegration of biofilm exposes many metabolites; some connection sugars and metabolic intermediates were significantly enriched. D-galactose exhibited the most dramatic upregulation (*p* < 0.01), followed by D-xylose (*p* < 0.01). These sugars are not major constituents of Psl but are abundant in the lipopolysaccharide (LPS) core oligosaccharide and cell wall polysaccharides of *P. aeruginosa*. Their enrichment indicates that Psl degradation exposes underlying cellular surface structures previously masked by the dense Psl overlayer. D-glucose-6-phosphate (G6P) accumulated significantly (*p* < 0.01), serving as the critical metabolic hub channeling released sugars into central carbon metabolism.

Psl structural core sugars were slightly depleted. D-mannose and D-glucose just showed slight changes; two sugars constitute the primary backbone of Psl polysaccharide, and their depletion implied that PslG mediated the endo-cleavage of the Psl glycan backbone. But there is no direct evidence. D-sorbitol, a key osmoprotectant sugar alcohol, showed the most pronounced depletion (*p* < 0.05), suggesting massive consumption to counteract osmotic stress during matrix swelling and dispersal. Maltose (*p* < 0.05) was significantly reduced, indicating a mobilization of intracellular carbon storage reserves and a breakdown of osmotic protective barriers as bacteria transitioned from the biofilm to the planktonic state. L-arabinose and D-glucuronic acid were also slightly decreased, consistent with the loss of auxiliary polysaccharide components cross-linked to the Psl network.

### 2.4. Untargeted Metabolomics Reveals Comprehensive Metabolic Reprogramming Toward Planktonic Proliferation

**Figure 4:**
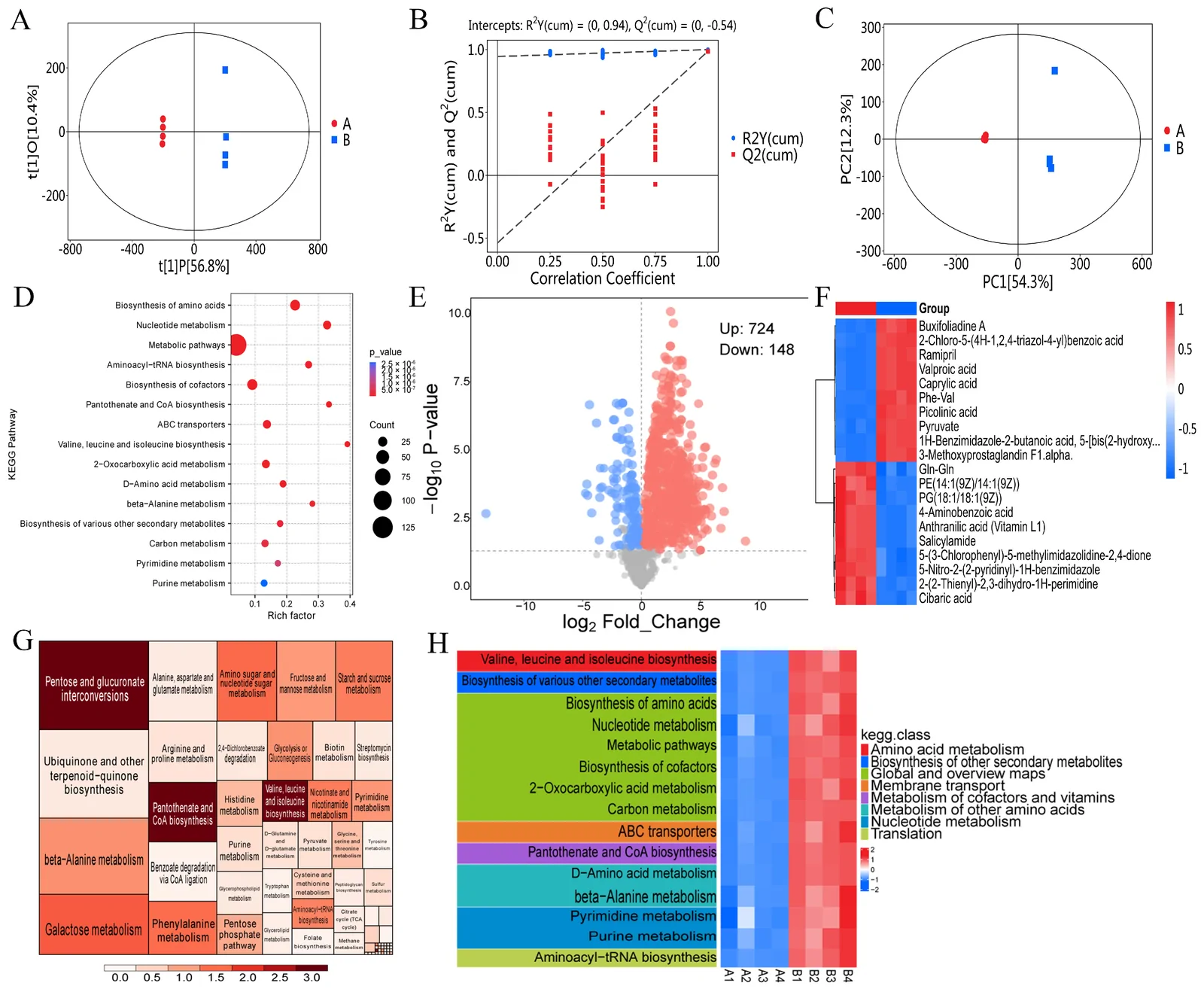
Untargeted metabolomics data display of biofilm (Group A, untreated biofilm) vs. PslG treatment (Group B, the biofilm treated by 50 nM PslG for 30 min). (**A**) Score scatter plot of OPLS-DA model for group A vs. B; (**B**) OPLS-DA for orthogonal coefficients and slopes; (**C**) the PCA scatter plots for group A vs. B; (**D**) KEGG enrichment for group A vs. B; (**E**) the volcano plot of differential metabolite screening shows the types of monosaccharides with differences, with blue representing downregulation and red representing upregulation; (**F**) heatmap of hierarchical clustering analysis for group A vs. B; (**G**) the metabolic pathways involved in differential metabolites; and (**H**) hierarchical clustering analysis heatmap of differential metabolites, with blue representing downregulation and red representing upregulation.

Untargeted metabolomics detected significant pathway enrichments (Figure 4). Top enriched KEGG pathways included: pentose and glucuronate interconversions, pantothenate and CoA biosynthesis, carbon metabolism, biosynthesis of amino acids, valine, leucine and isoleucine biosynthesis, nucleotide metabolism, and ABC transporters. These indicated activation of central carbon metabolism, energy production, and biosynthetic programs.

Principal component analysis (PCA) demonstrated a tight clustering of biological replicates (QC CV < 12%) and a clear trajectory of metabolic change: PC1 (54.3% variance) captured the transition from biofilm to dispersed state, while PC2 (12.3% variance) reflected intermediate stress responses. OPLS-DA modeling achieved robust class discrimination; the farther the lateral distance between samples, the greater the inter-group differences, and the closer the longitudinal distance, the better the intra-group repeatability (Figure 4A–C).

Pathway enrichment analysis using Metabo Analyst 5.0 revealed profound and coordinated activation of multiple metabolic modules (Figure 4D–H). The most significantly enriched pathways included:

Carbohydrate and energy metabolism: Pentose and glucuronate interconversions exhibited the highest enrichment significance, followed by fructose and mannose metabolism, galactose metabolism, carbon metabolism, and starch and sucrose metabolism. These enrichments directly mirror the glycomic substrate availability: xylose/galactose enrichment is consistent with activating pentose–glucuronate interconversions and galactose metabolism; and G6P accumulation channels carbon into glycolysis and the pentose phosphate pathway (PPP).

Energy production and cofactor synthesis: Pantothenate and CoA biosynthesis and ubiquinone and other terpenoid–quinone biosynthesis were significantly enriched. This indicates that acetyl-CoA—derived from glycolytic pyruvate via the pyruvate dehydrogenase complex; pyruvate shows an increase in content—is being funneled into the tricarboxylic acid (TCA) cycle and oxidative phosphorylation, generating ATP required for flagellar motility and biosynthetic activities.

Biosynthesis and growth metabolism: Biosynthesis of amino acids (overall), valine, leucine and isoleucine biosynthesis (BCAA), nucleotide metabolism, and aminoacyl-tRNA biosynthesis were all prominently activated. The BCAA pathway exhibited the highest differential abundance score, reflecting massive investment in protein synthesis infrastructure. Purine metabolism and pyrimidine metabolism were also significantly enriched, providing nucleotide precursors for DNA replication and transcription.

Membrane transport and environmental adaptation: ABC transporters showed significant enrichment, indicating upregulated nutrient scavenging capacity to internalize the sugars released from degraded Psl matrix before their diffusion into the bulk liquid.

Notably, no pathways associated with oxidative stress, cell death, or DNA damage response were significantly enriched, corroborating the non-lethal nature of PslG action observed in structural and viability assays.

## 3. Discussion

### 3.1. PslG as a Precision Molecular Scalpel: Mechanistic Specificity and Kinetic Efficiency

The fact that PslG achieves near-complete HHA-FITC signal loss within 30 min at nanomolar concentrations underscores its remarkable catalytic efficiency and positions Psl as the keystone structural element whose removal precipitates catastrophic matrix collapse [15,16,18]. The relatively modest depletion of L-rhamnose compared to mannose and glucose offers additional mechanistic insight. Rhamnose constitutes a minor component of Psl (~10–15%) and is predominantly located at the non-reducing termini of Psl oligosaccharide repeat units [12,14]. PslG’s endo-cleavage mode preferentially hydrolyzes internal glycosidic bonds within the Man-Glc-rich backbone, releasing large fragments enriched in terminal rhamnose that may rapidly diffuse away or remain as short stubs below detection thresholds. This differential cleavage pattern is consistent with the enzyme’s 32 Å-long active-site cleft, which accommodates extended polysaccharide chains for processive endo-attack rather than terminal exo-cleavage [15]. The increase in galactose raises the possibility that PslG may cross-react with other polysaccharides, which is worth further exploration.

### 3.2. The “Swelling Paradox”: Reinterpreting Biofilm Thickness Dynamics During Enzymatic Disruption

Perhaps the most counterintuitive finding of this study is the initial decrease followed by an increase in biofilm thickness during PslG-mediated disruption. Conventional intuition predicts that enzymatic matrix degradation should cause vertical compression or collapse. Our multi-omics data resolve this paradox through three convergent mechanisms.

First, osmotic swelling of the de-cross-linked matrix. Psl fibers function as the primary load-bearing elements of the biofilm matrix, maintaining structural integrity through hydrogen bonding, hydrophobic interactions, and entanglement with extracellular DNA and Pel polysaccharide [10,13]. PslG-mediated cleavage of these fibers effectively reduces the cross-link density of the hydrogel-like matrix, analogous to the swelling behavior of a chemically de-cross-linked polymer hydrogel. Upon removal of mechanical constraints, the hydrated EPS matrix absorbs water osmotically and expands in the vertical dimension, increasing the apparent thickness despite the loss of dry biomass.

Second, bacterial vertical redistribution into previously inaccessible space. In intact biofilms, dense Psl networks physically confine bacterial microcolonies to the basal region, creating a stratified architecture with an apical “Psl cap” and basal “cell layer” [14]. Psl removal eliminates this physical barrier, allowing bacteria to redistribute into the expanded matrix volume and even escape into the overlying liquid. The CLSM data showing SYTO 9 fluorescence extending to the apical region by 30 min directly visualizes this redistribution. The measured thickness increase thus reflects the expanded spatial occupation of the same biomass, not growth.

Third, exposure of underlying cellular structures increases apparent optical density. The glycomic enrichment of galactose, xylose, and galacturonic acid—sugars abundant in LPS core oligosaccharides and cell wall polysaccharides but not in Psl—demonstrates that Psl degradation exposes previously concealed cellular surface structures [7,25]. These newly exposed glycoconjugates contribute to the fluorescent signal within the expanded volume, further augmenting the measured thickness.

This reinterpretation has critical implications for biofilm quantification: thickness and biomass are not synonymous. Traditional endpoint assays (crystal violet, dry weight) measure total organic material, while confocal thickness measures spatial extent. Our data demonstrate that enzymatic disruption can increase spatial extent while decreasing structural integrity—a distinction that single-parameter assays cannot capture.

### 3.3. From Structural Collapse to Metabolic Renaissance: The Glycomic–Metabolomic Axis

The integration of glycomic and metabolomic data reveals that PslG treatment transforms the biofilm matrix from a protective prison into a nutritional buffet. The released and exposed sugars do not simply diffuse away; rather, they are efficiently scavenged and channeled into central carbon metabolism, fueling a comprehensive metabolic renaissance.

The G6P nexus: a metabolic switchboard. The significant accumulation of glucose-6-phosphate (G6P) serves as the critical metabolic hub integrating extracellular sugar availability with intracellular metabolic flux. G6P occupies a unique position at the crossroads of glycolysis (energy production), the pentose phosphate pathway (PPP; nucleotide synthesis and NADPH generation), and glycogen/storage polysaccharide synthesis [26,27]. Its accumulation indicates that the rate of sugar phosphorylation by hexokinases exceeds the downstream processing capacity of glycolysis and PPP—a metabolic “traffic jam” reflecting sudden carbon abundance.

The pentose phosphate pathway: powering biosynthesis and antioxidant defense. The dramatic upregulation of xylose and the significant enrichment of pentose and glucuronate interconversions indicate robust PPP activation. Xylose enters bacterial metabolism via xylose isomerase and xylulokinase, generating xylulose-5-phosphate that feeds directly into the non-oxidative phase of PPP [28]. PPP activation serves dual functions: (i) generating ribose-5-phosphate for nucleotide and nucleic acid synthesis, supporting the concurrent enrichment of nucleotide metabolism and aminoacyl-tRNA biosynthesis; and (ii) producing NADPH, the essential reducing equivalent for fatty acid biosynthesis, antioxidant defense (glutathione reduction), and the thioredoxin system. The absence of oxidative stress pathway enrichment in metabolomics, despite massive matrix disruption, likely reflects the protective NADPH supply from activated PPP.

CoA and ubiquinone synthesis: energizing the planktonic transition. The significant enrichment of pantothenate and CoA biosynthesis and ubiquinone biosynthesis indicates that dispersed cells are investing heavily in aerobic respiration infrastructure. CoA is the central acyl carrier in metabolism; its synthesis from pantothenate (vitamin B5) is essential for the pyruvate dehydrogenase reaction that generates acetyl-CoA for TCA cycle entry [29]. Ubiquinone (coenzyme Q) serves as the electron shuttle between Complex I/II and Complex III in the respiratory chain. The coordinated upregulation of these pathways suggests that dispersed cells are transitioning from the oxygen-limited, fermentative metabolism typical of biofilm microcolonies to the fully aerobic respiration characteristic of planktonic growth [30,31].

Branched-chain amino acid synthesis: preparing for proliferation. The BCAA pathway (valine, leucine, isoleucine) exhibited the highest differential abundance score among all metabolic pathways. BCAAs are essential precursors for protein synthesis and are also incorporated into membrane lipid modification (branched-chain fatty acids) and quorum-sensing signal molecules [21]. Their massive upregulation, combined with general amino acid biosynthesis and aminoacyl-tRNA synthesis, indicates that dispersed cells are not merely surviving but are actively preparing for cell division—a metabolic state diametrically opposed to the dormant, stress-tolerant phenotype of biofilm-embedded cells.

### 3.4. Non-Lethal Dispersal: Therapeutic Implications and Biological Significance

Stable PI fluorescence suggests that PslG treatment did not cause a marked increase in membrane permeability under the examined imaging conditions, establishing that PslG-mediated biofilm disruption is fundamentally non-lethal. This distinguishes PslG from conventional anti-biofilm strategies such as DNase I (which can induce DNA damage responses), dispersin B (which may expose bacteria to immune attack), or antibiotic treatment (which selects for persisters) [23,24].

Previous studies reported that PslG-mediated matrix disruption increased antibiotic penetration and host-mediated clearance and improved bacterial clearance in infection models [16,18,19]. These findings support reduced biofilm-associated persistence but do not demonstrate that the present treatment suppresses intrinsic virulence-factor expression. Previous PslGh studies also reported no detectable toxicity toward human lung fibroblasts, human red blood cells, or neutrophils and no impairment of porcine wound healing [16,19]. The present study did not conduct new cytotoxicity, hemolysis, or infection assays; consequently, the cited evidence is described as preliminary biocompatibility support rather than a complete safety assessment of the current formulation.

From a therapeutic perspective, this non-lethal dispersal phenotype is a double-edged sword with strategic advantages. On one hand, PslG alone does not kill bacteria; on the other hand, it releases metabolically active, membrane-intact cells that have regained antibiotic susceptibility. The metabolic renaissance observed in our data—characterized by active respiration, biosynthesis, and nutrient uptake—renders these dispersed cells particularly vulnerable to antibiotics that target active metabolic processes (e.g., fluoroquinolones, aminoglycosides) rather than dormant cells [19]. This supports the “Trojan Horse” therapeutic strategy: PslG acts as the horse that breaches the biofilm fortress, delivering vulnerable bacteria to conventional antibiotics.

Furthermore, the absence of oxidative stress and cell-death pathways suggests that PslG does not trigger the stress responses that often lead to persister formation or adaptive resistance. This is in stark contrast to bactericidal agents, which at sub-MIC concentrations can induce recalcitrance through SOS responses, stringent response, or biofilm reformation [32].

### 3.5. Limitations and Future Directions

Several limitations of this study should be acknowledged. First, our in vitro static biofilm model lacks the dynamic fluid shear, host immune factors, and polymicrobial competition present in natural infection sites. The observed “swelling” behavior may differ under flow conditions where hydrodynamic forces could accelerate bacterial washout. Second, while our metabolomic coverage is comprehensive for central metabolism, specialized signaling molecules such as c-di-GMP, ppGpp, and quorum sensing autoinducers were below the detection limit of our untargeted LC-MS/MS method. Moreover, based on the thickness of the biofilm and the presence of live bacteria inside, we cannot guarantee the distinction between sugars still present in the degraded extracellular matrix and sugars that have already been internalized and phosphorylated, such as G6P, even when washed with PBS; targeted metabolomics would provide definitive confirmation of their dynamics. Third, the thickness increase we observed is based on confocal Z-stack projections; advanced techniques such as optical coherence tomography or light-sheet microscopy could provide more accurate volumetric reconstructions without phototoxicity concerns.

Future studies should extend this multi-omics framework to: (i) clinical *P. aeruginosa* isolates with diverse mucoidy and resistance profiles; (ii) in vivo infection models to validate the dispersal-antibiotic synergy hypothesis; (iii) proteomic analysis to identify the specific ABC transporters and metabolic enzymes upregulated during dispersal; and (iv) isotope tracing and functional assays of respiration, ATP, motility, sugar uptake, and proliferation, and longitudinal assessment of regrowth, biofilm reformation, and resistance evolution. A recent transcriptomic–metabolomic study showed that RpoS and Crp coordinate metabolic adaptation during acid stress in *Escherichia coli* [33]. This study provides conceptual support for global regulation of bacterial metabolic adaptation but does not directly establish RpoS involvement in PslG-treated *P. aeruginosa*; it can provide reference value for further in-depth research in the future.

## 4. Materials and Methods

### 4.1. Bacterial Strains and Culture Conditions

*Pseudomonas aeruginosa* PAO1 (wild type) was used as the biofilm formation strain throughout this study. For routine cultivation, bacteria were streaked onto LB agar plates and incubated at 37 °C for 16–18 h. Single colonies were inoculated into 5 mL LB broth and grown overnight (37 °C, 200 rpm) to obtain starter cultures.

Biofilm formation was conducted in Jensen minimal medium (NaCl 5 g, K_2_HPO_4_ 2.51 g/L, L-glutamate monosodium salt 15.56 g/L, valine 2.81 g/L, phenylalanine 1.32 g/L, glucose 13.87 g/L, MgSO_4_ 7H_2_O 0.165 g/L, CaCl_2_ 2H_2_O 0.105 mg/L, FeSO_4_ 7H_2_O 5.5 μg/L, ZnSO_4_ 7H_2_O 12 μg/L, and deionized water supplemented to 1 L. Starter cultures were diluted to an optical density at 600 nm (OD_600_) of 0.01 in fresh Jensen medium and inoculated into 96-well polystyrene plates (100 μL/well). Static biofilms were cultivated at 37 °C without agitation for 24 h to achieve mature biofilm development.

### 4.2. Biofilm Disruption

Mature 24 h biofilms were subjected to controlled enzymatic disruption using PslG glycan hydrolase (PslG), a specific Psl polysaccharide-degrading enzyme. Control groups included: (a) untreated biofilms incubated in buffer alone (negative control) and (b) biofilms treated with different concentrations of PslG (1–1000 nM). The remaining biofilm biomass was determined by CV staining assay as described in the biofilm formation assay. CV staining experiments were performed in biological triplicates with technical duplicates for each analytical endpoint.

### 4.3. Biomass Quantification

Crystal violet staining was employed for total biomass assessment. Biofilms were washed twice with phosphate-buffered saline (PBS, pH 7.4) to remove non-adherent cells and stained with 0.1% (*w*/*v*) crystal violet for 15 min at room temperature. Excess dye was removed by washing with distilled water, and bound crystal violet was solubilized in 40% (*v*/*v*) acetic acid with agitation at 200 rpm for 15 min. Absorbance was measured at 560 nm using a microplate reader. Significance was determined using Student’s *t*-test.

### 4.4. Monosaccharide, Disaccharide and Sugar Derivative Composition Analysis

Targeted carbohydrate analysis was performed on whole biofilm after PBS washing; Group A consisted of untreated *P. aeruginosa* biofilms, and Group B consisted of PBS resuspensions of PslG treated with a concentration of 50 nM for 30 min to disintegrate the biofilms. Each group consisted of four biological replicates. Sample processing and data reporting were issued by Shanghai biotree biomedical technology co., ltd. Approximately 50 mg of lyophilized sample was extracted with 50% methanol–water, homogenized by bead grinding, and subjected to ultrasonic-assisted extraction. After centrifugation, the supernatant was collected and dried under vacuum. The residue was sequentially derivatized with 80 μL of 20 mg/mL methoxyamine hydrochloride in pyridine at 60 °C for 60 min (oximation), followed by 100 μL of BSTFA-TMCS (99:1, *v*/*v*) at 70 °C for 90 min (silylation). Following centrifugation, the supernatant was transferred to an autosampler vial for analysis. Chromatographic separation was performed on an UHPLC system (Vanquish, Thermo Fisher Scientific, Langenselbold, Germany) with a Waters ACQUITYUPLC BEH Amide (2.1 mm × 50 mm, 1.7 μm) coupled to Orbitrap Exploris 120 mass spectrometer (Orbitrap MS, Thermo, Germany). Helium was employed as the carrier gas at a flow rate of 1.0 mL/min. The injector, transfer line, and ion source temperatures were set at 280 °C, 320 °C, and 230 °C, respectively. Mass spectra were acquired over the range *m*/*z* 85–500 at a scan rate of 10 spectra per second. Monosaccharides, disaccharides, and sugar derivatives were identified by matching retention times with 19 authentic carbohydrate standards and quantified using external calibration curves (R^2^ > 0.99). Statistical analysis: Multivariate analysis was conducted in SIMCA18.0.1 software (Sartorius Stedim Data Analytics AB, Umea, Sweden). Data were log-transformed, Pareto-scaled, and analyzed by principal component analysis (PCA) for quality assessment and orthogonal partial least squares discriminant analysis (OPLS-DA) for class discrimination. Model validity was assessed by cross-validation and permutation testing (*n* = 200). Significance was determined using Student’s *t*-test (“ns”, not significant, * *p* < 0.05; ** *p* < 0.01; *** *p* < 0.001; **** *p* < 0.0001).

### 4.5. Untargeted Metabolomics

Untargeted metabolomics was performed on whole biofilm after PBS washing, Group A consisted of untreated *P. aeruginosa* biofilms, and Group B consisted of PBS resuspensions of PslG treated with a concentration of 50 nM for 30 min to disintegrate the biofilms. Each group consisted of four biological replicates. Sample processing and data reporting were issued by Shanghai biotree biomedical technology co., ltd. Metabolite extraction employed a modified Bligh–Dyer method. Biofilms were rapidly quenched by direct addition of −40 °C cold 60% methanol (2 mL) to halt metabolic activity. Cell suspensions were transferred to pre-cooled tubes, vortexed for 30 s, and subjected to three freeze–thaw cycles (liquid nitrogen/37 °C water bath) to enhance extraction efficiency. Chloroform (1 mL) and water (1 mL) were added to achieve phase separation (methanol:chloroform:water, 2:1:1 *v*/*v*/*v*), vortexed for 2 min, and centrifuged (14,000× *g*, 10 min, 4 °C). The upper polar phase was collected, dried in a vacuum concentrator, and stored at −80 °C pending analysis.

LC-MS/MS analysis was performed on a Q Exactive HF-X hybrid quadrupole-Orbitrap mass spectrometer coupled to a Vanquish UHPLC system. Dried extracts were reconstituted in 100 μL LC-MS grade water and centrifuged (14,000× *g*, 5 min) to remove particulates.

Chromatographic separation employed a ZIC-pHILIC column (150 × 2.1 mm, 5 μm) maintained at 40 °C for polar metabolite analysis. Mobile phases consisted of (A) 20 mM ammonium carbonate in water (pH 9.2) and (B) acetonitrile. The gradient program was: 0–2 min, 90% B; 2–17 min, 90–40% B; 17–18 min, 40% B; 18–18.5 min, 40–90% B; and 18.5–22 min, 90% B for column re-equilibration. Flow rate was 0.3 mL/min, and injection volume was 5 μL.

Mass spectrometry parameters: Electrospray ionization (ESI) in polarity switching mode; spray voltage 3.5 kV (positive) and 3.2 kV (negative); capillary temperature 320 °C; sheath gas flow rate 35 arb; auxiliary gas flow rate 10 arb; and probe heater temperature 350 °C. Full MS scans were acquired at resolution 70,000 (*m*/*z* 70–1050, AGC target 3 × 10^6^, maximum IT 100 ms). Data-dependent MS/MS (ddMS^2^) employed stepped normalized collision energies (20, 40, 60 eV) with resolution 17,500, isolation window 1.0 *m*/*z*, AGC target 1 × 10^5^, maximum IT 50 ms, and dynamic exclusion 15 s.

Data processing: Raw LC-MS data were processed using Compound Discoverer 3.2 with the following workflow: retention time alignment (adaptive curve correction), peak detection (minimum intensity 1 × 10^5^, S/N ratio > 3), compound grouping (mass tolerance 5 ppm, RT tolerance 0.2 min), and gap filling. Peak areas were normalized by probabilistic quotient normalization (PQN) to correct for technical variation.

Compound identification utilized mzCloud, mzVault in-house libraries, and KEGG/HMDB database matching based on accurate mass (<5 ppm), isotopic pattern, and MS/MS spectral similarity (cosine score > 0.7). Metabolite annotation confidence followed MSI levels: Level 1 (authentic standard match), Level 2 (MS/MS match), Level 3 (accurate mass and predicted formula), Level 4 (accurate mass only).

Quality control and assurance: A pooled quality control (QC) sample prepared by combining equal aliquots (10 μL) from all experimental samples was injected every 10 samples to monitor system stability. Blank injections (extraction solvent) were interspersed to detect background contamination. System suitability was assessed by monitoring retention time stability (CV < 3%), peak area reproducibility (CV < 15% for QC features), and mass accuracy (<5 ppm).

Statistical analysis: Multivariate analysis was conducted in SIMCA18.0.1 software (Sartorius Stedim Data Analytics AB, Umea, Sweden). Data were log-transformed, Pareto-scaled, and analyzed by principal component analysis (PCA) for quality assessment and orthogonal partial least squares discriminant analysis (OPLS-DA) for class discrimination. Model validity was assessed by cross-validation and permutation testing (*n* = 200). Significance was determined using Student’s *t*-test (“ns”, not significant, * *p* < 0.05; ** *p* < 0.01; *** *p* < 0.001; **** *p* < 0.0001).

Pathway enrichment analysis was performed using MetaboAnalyst 5.0 with pathway topology analysis (relative-betweenness centrality) against the KEGG pathway library for *P. aeruginosa* (pae). Enrichment significance was determined by Fisher’s exact test (*p* < 0.05) with FDR correction.

### 4.6. Three-Dimensional Structure Imaging

Confocal laser scanning microscopy (CLSM) was performed on a Zeiss LSM 900 (Carl Zeiss Microscopy GmbH, Jena, Germany) equipped with 405 nm, 488 nm, 561 nm, and 633 nm laser lines. Biofilms were cultivated in 1 × 1 × 4 cm chamber (Lab-Tek II, Chambered #1.5 German Coverglass system-155382, Thermo Fisher Scientific, Rochester, NY, USA) for optimal optical properties.

Fluorescent staining protocols:

LIVE/DEAD staining was performed using the BacLight Bacterial Viability Kit (Molecular Probes, Invitrogen, Eugene, OR, USA). Biofilms were washed three times with PBS and incubated with 5 μM SYTO 9 and 5 μM propidium iodide (PI) in PBS for 20–30 min at room temperature in the dark. Imaging was performed on a confocal laser scanning microscope with 480/500 nm for SYTO 9 stain and 490/635 nm for PI.

Psl polysaccharide was labeled with 100 μg/mL HHA-FITC in PBS for 30 min at room temperature in the dark. After washing three times with PBS, biofilms were stained with 5 μM propidium iodide (PI) for 20 min at room temperature in the dark, followed by two PBS washes. Imaging was performed on a confocal laser scanning microscope with 488 nm excitation (500–530 nm emission) for HHA-FITC and 561 nm excitation (620–660 nm emission) for PI.

Image acquisition: Z-stack images were collected using a 20×/0.8 NA Plan-Apochromat objective (large field overview) and 63×/1.4 NA Oil Plan-Apochromat objective (high-resolution detail) of a Zeiss LSM 900 (Carl Zeiss Microscopy GmbH, Jena, Germany). Pinhole diameter was set to 1 Airy unit. Z-step size was optimized at 1.0 μm based on Nyquist sampling criteria. Pixel dimensions were 0.099 μm × 0.099 μm (20×) or 0.10 μm × 0.10 μm (63×) with 12-bit dynamic range.

Three-dimensional reconstruction and analysis: Deconvolved images were rendered in ZEN 3.14 of a Zeiss LSM 900 (Carl Zeiss Microscopy GmbH, Jena, Germany). The biofilm biomass and thickness were quantified by COMSTAT software version 2.1 [20].

Quantitative structural parameters:

Biofilm thickness (μm): Maximum-intensity projection along *Z*-axis; average thickness calculated from 10 random line profiles per image.

## 5. Conclusions

This study integrated glycomic, metabolomic, and structural imaging data to decode the mechanism of PslG-mediated *P. aeruginosa* biofilm disruption. PslG markedly reduced HHA-FITC-detectable Psl signal, while biofilm thickness showed rapid thinning followed by partial recovery that remained below baseline. D-glucose-6-phosphate (G6P), xylose and D-galactose significantly increased. Our findings revealed that PslG induces rapid Psl depolymerization, non-lethal bacterial dispersal with counterintuitive matrix swelling and vertical redistribution, and comprehensive metabolic reprogramming toward aerobic respiration and biosynthesis, including pentose and glucuronate interconversions, pantothenate and CoA biosynthesis, carbon metabolism, biosynthesis of amino acids, nucleotide metabolism, and ABC transporters. These indicated the activation of central carbon metabolism, energy production, and biosynthetic programs. The established Cascade Disruption Model resolves the apparent paradox of thickness increase during disruption and provides a mechanistic framework for understanding how enzymatic matrix degradation translates into cellular behavioral change. By targeting the keystone Psl scaffold and triggering the bacterium’s own dispersal programs, PslG represents a precision anti-biofilm strategy that converts recalcitrant biofilm cells into antibiotic-susceptible planktonic cells without inducing stress-mediated resistance. These insights pave the way for rational design of next-generation combination therapies against biofilm-associated infections.

## Data Availability

The original contributions presented in this study are included in the article. Further inquiries can be directed to the corresponding author.
